# Nanotechnology in Lung Cancer Therapeutics: A Narrative Review

**DOI:** 10.7759/cureus.34245

**Published:** 2023-01-26

**Authors:** Vaibhav Koutu, Manish Gupta, Saikat Das, Deepak K Rawat, Vipin Kharade, Rajesh K Pasricha

**Affiliations:** 1 Radiotherapy, All India Institute of Medical Sciences, Bhopal, Bhopal, IND

**Keywords:** therapeutics, drug delivery, nanomedicines, nanomaterials, lung cancer

## Abstract

To date, cancer continues to be one of the biggest challenges for medical science. Nanotechnology has enabled us to overcome some of the limitations of conventional treatment in lung cancer therapeutics. Recently, US Food and Drug Administration (FDA) has approved certain nanomedicines for clinical administration against lung cancer. This article presents a narrative review of approved nanomedicines in lung cancer with a special focus on the results of recently concluded and ongoing clinical trials. The limitations associated with using nanomaterials as anti-lung cancer therapeutic agents and the possible mechanisms to overcome these limitations are also discussed.

## Introduction and background

Cancer has been the leading cause of death worldwide, accounting for nearly 10 million deaths in 2020 which means nearly one in every six deaths. Out of the different types of known cancers to date, only lung cancer has been reported to be the reason for nearly 1.80 million deaths globally in 2020-2021 [1,2].

With a considerably high mortality rate of one out of every five deaths worldwide due to cancer, lung cancer is considered one of the most challenging cancers to manage [1]. According to a recent World Health Organization (WHO) report, lung cancer is the sixth most common cause of mortality contributing to nearly 1.8% of total deaths. To date, early diagnosis and treatment of lung cancer remain a challenge [3]. Due to drastic changes in environmental conditions, unplanned and unhealthy lifestyles, and exposure to numerous pollutants and radiations, it is estimated that the number of lung cancer patients could increase by nearly 50% over the next 20 years [4]. There is a lack of reciprocity between the progress made in diagnosing cancer at a molecular level and clinically relevant translational advances, which infers that still more understanding about lung cancer is required for effective therapy [5,6].

Out of the conventional therapeutic techniques (chemotherapy, radiotherapy, biological therapy, and surgery), the percentage of patients presenting in the early stage amenable to local treatment like resection and/or radiation is less due to diagnosis at an advanced stage or metastasis at presentation. Therefore, systemic therapy remains a cornerstone in the treatment of lung cancer. In the past few years, the focus of researchers has significantly shifted toward the development of new molecules and techniques of drug delivery to enhance efficacy with reduced toxicity [7,8].

Nanotechnology is an enabling technology that involves the utilization of nanomaterials for targeted therapy. Nanoparticles exhibit certain properties to enhance reactive areas as well as across cell or tissue junction barriers, which is nearly 150-200 µm [5]. Due to their multifaceted properties and features, nanoparticles have emerged as potential candidates to overcome the biological and chemical barriers inside the human body, thus permitting increased diagnostic and therapeutic efficacy with lower invasiveness and higher biocompatibility [5,6]. In this article, we will critically analyze the FDA-approved nanomedicines relevant to lung cancer and their clinical implications as evident from the results of the clinical trials.

## Review

Recent advances in lung cancer therapeutics in a clinical context

Despite numerous advances that have been made in the field of oncological diagnostics and therapeutics sciences, clinical management of lung cancer still continues to be a challenge. Some of the major factors contributing to the unsolved puzzle of lung cancer are delayed diagnosis, the propensity of metastasis, multidrug resistance, and unpredictable tumor environment, which ultimately causes hurdles to therapeutic strategies. Despite advancements in molecular imaging, personalized therapeutics are in their infancy due to the unavailability of cancer-specific nano-biomarkers, which would provide very precise information and detection about the outbreak of cancer at very low concentrations [4]. In the field of cancer therapeutics also, certain advancements have been made like combination chemotherapy, nanoparticle-assisted chemotherapy, immunology-based therapy, chemo combined with radiotherapy, biological therapy, and others; however, the count of positive outcomes of these advanced therapeutic technologies is still far behind the number of cases or deaths recorded annually. This shows that the current scenario requires many-fold advancements in the field of lung cancer therapy. The advent of nanotechnology has provided new hope in the field of cancer therapeutics.

Nanoparticles are usually not directly utilized as anticancer medicines; instead, they are used in conjunction with conventional drugs or active ingredients to improve their cell permeability and efficiency, and also to reduce the side effects and morbidity of advanced cancer therapies [9]. Conventionally, the active ingredient is either encapsulated to increase the target‘s ability and travel time in the bloodstream (nanovesicles) or bonded covalently to the nanomaterial, which provides a precise number of therapeutic molecules for each nanoparticle [5]. It is a well-elucidated fact that most anticancer drugs are hydrophobic, resulting in many challenges for physiological uptake; hence, encapsulating them or covalently bonding them with nanoparticles assists in the controlled release of the drug to ensure specific toxicity to the tumor tissue sparing the normal tissue and prevent rapid clearance from the bloodstream, thus enhancing their bioavailability [10,11]. Table 1 shows the various types and platforms of nanoparticles utilized in anticancer therapy.

**Table 1:** Types of nanoparticles to be used as nanomedicines Source: References [7,8].

Types of Nanomaterials			
Lipid-based nanocarriers	Inorganic nanoparticles	Drug conjugates	Polymer-based nanocarriers
Solid lipid nanoparticles	Silica nanoparticle	Antibody-drug conjugate	Polymeric micelle
Liposome	Metal nanoparticle	Polymer-drug conjugate	Nanoparticle albumin-bound (Nab) technology
Encapsulated liposome	Metal-oxide nanoparticle	Polymer-protein conjugate	Polymeric nanoparticle

Systemic therapy in lung cancer

Systemic therapy in lung cancer includes chemotherapy, biological therapy, and immunotherapy. It has been clinically and scientifically proven that in lung cancer, the identification of oncogenic driver mutations (in genes such as epidermal growth factor receptor [EGFR], anaplastic large-cell lymphoma kinase [ALK], c-ros oncogene 1 [ROS1], and vascular endothelial growth factor receptor [VEGFR]) has successfully led to the development of small drug molecules that are specific to the products of these mutated genes with varying degrees of clinical responsiveness [6,7].

Several factors are responsible for the limitations in lung cancer therapeutics, which include lack of diagnosis at an early stage, patient-specific unpredictable inter- and intra-tumor heterogeneity, lack of knowledge about the tumor environment and its genomics, and the problem of drug delivery. The criticality of the tumor microenvironment and its various components (infiltrating immune cells and mesenchymal cells along with its close association with the extracellular matrix and vasculature) have been reviewed by Chen et al. [12]. The literature has discussed the variety of tumor microenvironments in the population depending on the genetic constitution, vasculature, and immunity [13-15].

Among the list of clinically approved drugs available for the therapy of cancers worldwide, FDA has approved a very small number of therapeutic drugs for the treatment of lung cancer [16-19]. This is attributed to the absence of biostability of therapeutic and targeting moieties, very limited knowledge about molecular targets in lung cancer, failure of enhanced permeation retention (EPR) phenomenon, and active targeting in complex biological environments [4,20,21]. Table 2 enlists the list of drugs commonly used for lung cancer therapy [3,4,22-61].

**Table 2:** List of drugs approved by FDA for lung cancer therapy FDA: Food and Drug Administration.

Types of Agents	Name of Drug/Medicine	References
Chemotherapeutic agents	Doxorubicin	[3,4,22]
	Methotrexate	[22,23]
	Etoposide phosphate	[4,24]
	Mechlorethamine	[24,25]
	Topotecan	[26]
	Albumin-stabilized nanoparticle formulation of paclitaxel	[3,27]
	Carboplatin	[28]
	Atezolizumab with nab-paclitaxel and carboplatin	[29]
	Nivolumab	[30]
	Docetaxel	[31]
	Ramucirumab	[32]
	Capmatinib	[33]
	Gemcitabine	[34]
	Mechlorethamine	[24,25]
	Methotrexate	[22,23]
	Paclitaxel	[35]
	Pemetrexed disodium	[36]
	Vinorelbine tartrate	[37]
Biologically targeted therapeutic agents	Everolimus	[38]
	Afatinib	[39]
	Alectinib	[40]
	Brigatinib	[41]
	Ceritinib	[42]
	Crizotinib	[43]
	Dacomitinib	[44]
	Entrectinib	[45]
	Erlotinib HCl	[46,47]
	Gefitinib	[48,49]
	Osimertinib mesylate	[50,51]
	Trametinib	[52]
Immunotherapeutic agents	Atezolizumab	[53]
	Pembrolizumab	[54,55]
	Nivolumab	[56]
	Bevacizumab	[57,58]
	Durvalumab	[59]
	Necitumumab	[60]
	Ramucirumab	[61]

Advantages of nanoparticles in cancer therapeutics

Most anticancer drugs are hydrophobic, which infers that they can easily penetrate the cellular membrane; however, on the contrary, their hydrophobicity usually leads to accumulation and embolism [5]. To overcome this drawback, these anticancer drugs can conveniently be formulated in nanoparticles, which facilitate the transport of the drugs through the bloodstream, thus improving the targetability and bioavailability and confiscating their removal from the body [3,62]. In addition, at the nanoscale, the properties of materials get modified due to the quantum size effect. Nanomaterials have various advantages compared to their bulk counterpart. These include a large surface-to-volume ratio, high surface energy, self-assembly, adaptable crystal structure, physiochemical stability, and controllable morphology. Under these unique properties, nanomaterials become the most potential candidate in the field of pharmacology and medicines [63,64]. Several other exclusive properties like target-specific (tumor) cytotoxicity, improved bioavailability, sustained drug release, and better pharmacokinetics could also be imparted to nanomaterials, which enables them to be used in the medicinal field.

Cytotoxicity

Surface charge (zeta potential) and large surface area of nanomaterials are the key factors contributing to the enhancement of site-specific cytotoxicity [65]. Tumor-specific toxicity or targeted toxicity of nanomaterials can be achieved by either loading corresponding drug molecules on the surface of nanomaterials or by encapsulating the drug molecule with targeted biomolecule-coated nanomaterials. This type of surface modification facilitates the nanomaterials to be utilized as nanocarriers to deliver drugs to the specifically targeted tumor sites, thereby reducing various types of side effects like drug accumulation or cellular toxicity [65-67].

Bioavailability

The rate and extent to which the active moiety of a drug is absorbed from a drug product and reaches body circulation is referred to as bioavailability [68,69]. Low or limited bioavailability of anticancer drugs associated with a wide variability is one of the common drawbacks of conventional drug-delivery techniques of cancer therapeutics [70]. On the other hand, nanomaterials provide better control and alteration in the bioavailability of administered drugs because depending upon the requirement, the bioavailability of the nanomaterials can be enhanced by altering their physicochemical and surface properties [69]. The surface characteristics of nanomaterials greatly influence their bioavailability and half-life. For this purpose, nanomaterials are intentionally modified to become hydrophilic, which increases the time of drugs in circulation and enhances their penetration and accumulation in tumors [7,69].

Sustained Drug Release

One of the major drawbacks of conventional drug-delivery mechanisms in anticancer therapeutics is the rapid and complete release of drug molecules in the patient's body or circulatory system immediately after administration. This generally leads to various types of toxicities in the body including high drug concentration in the blood plasma, risk of overmedication, drug accumulation in the non-desired sites or organs, and poor absorption of drugs in the tumor site. Also, it is associated with the frequent dosing of drugs in regular time intervals, failing to which reduces the efficacy of anticancer drugs. This drawback can be eliminated or greatly reduced by adopting a sustained drug release mechanism, the main objective of which is to improve the stability, bioavailability, and pharmacokinetics of anticancer drugs [71,72]. Sustained drug release can be defined as any drug or dosage form modification that prolongs the therapeutic activity of the drug [73].

To precisely control the drug release for a longer time, various nanomaterials have been developed and designed to recognize and handle subtle environmental changes associated with the tumor microenvironment (TME) and tumor cells like pH, redox state, and enzymes [74]. Some stimuli-responsive nanomaterials have also been developed, which can be activated by external stimuli (heat, light, magnetic field, or ultrasound) to trigger the release of the adsorbed or encapsulated drugs.

Pharmacokinetics

Pharmacokinetics of the release mechanism of the conventional anticancer agent comprises certain factors such as drug dose and release rate, drug absorption rate including the elimination half-life, the biological half-life of the drug, metabolism of the drug, drug-protein binding, and dosage form index [63]. The pharmacokinetic properties of nanomaterials are drastically altered by the reduction in the particle size as well as the modification in surface properties. Chiang et al. [5] discussed that for pharmacokinetic properties in nanomaterials, the optimal size should be around 100 nm in a hydrodynamic diameter. Recent studies have also proven that nanomaterials as drug carriers or encapsulation agents have shown improved pharmacokinetics and reduced side effects as compared to the parent drug [6].

Nanomedicines in anticancer therapy

Nanoparticle-based targeted drug-delivery systems utilize the various site-specific factors in the desired tumor site such as products of genetic mutations, enzyme concentrations, pH, vasculature, or other factors in the tumor microenvironment. The stimuli-responsive drug-delivery systems work on the release of anticancer drugs under the presence of unique stimulating factors [11,12]. These stimulating factors can be either "endogenous," which includes pH, redox agents, and enzymes, or "exogenous," which includes light, ultrasound, and magnetic field. Optimization of these factors improves the specific uptake of nanoparticles into tumor tissue [75].

Currently, about 20 commercial nanomaterial-based drugs for cancer therapeutics are either approved or in the process of being approved by the FDA, out of which around 85% are based on liposomes and polymer micelles [11]. Along with the liposomes and polymers, the focus is also being provided on loading biomolecules such as proteins, peptides, DNA, RNA, and other materials into nanoparticles to increase the efficiency of the drugs. Table 3 shows the list of some FDA-approved nanomedicines for anticancer therapeutics.

**Table 3:** List of some FDA-approved nanomedicines for anticancer therapeutics

Name of Nano-formulation	Active Drug Loaded	Nanomaterial Type	Cancer Therapy	Year of Approval	References
Doxil/Calex	Doxorubicin	Liposome (PEGylated)	HIV-associated Kaposi’s sarcoma, ovarian cancer, metastatic breast cancer, and multiple myeloma	1995	[3]
DaunoXome/Galen	Daunorubicin	Liposome (PEGylated)	HIV-associated Kaposi’s sarcoma	1996	[5]
DepoCyt	Cytosine arabinoside (cytarabine)	Liposome	Neoplastic meningitis	1999	[3]
Eligard	Leuprolide acetate	Polymer	Prostate cancer	2002	[10]
Abraxane	Paclitaxel	Nanoparticle albumin-bound (Nab)	Advanced non-small cell lung cancer, metastatic pancreatic cancer, and metastatic breast cancer	2005	[8,9]
Marqibo	Vincristine	Liposome (non-PEGylated)	Philadelphia chromosome-negative acute lymphoblastic leukemia	2012	[5,8]
Abraxane	Paclitaxel + Gemcitabine	Nab	Metastatic pancreatic cancer	2013	[8]
Onivyde/MM-398/Merrimack	Irinotecan	Liposome (PEGylated)	Metastatic pancreatic cancer (second line)	2015	[3,5]

Clinical trials related to nanoparticle-based delivery system

Untch et al. [13] conducted the phase-III clinical trial to improve disease-free survival in early breast cancer (BC) using GBG 69-GeparSepto. Their study demonstrated that weekly nanoparticle albumin-bound (NAB)-paclitaxel has significantly improved the pathologic complete remission rate when compared to that of weekly solvent-based (sb) paclitaxel followed by epirubicin plus cyclophosphamide as neoadjuvant treatment in patients with primary BC. The outcome of their randomized phase-III neoadjuvant GeparSepto trial study showed a significantly enhanced pathologic complete response (pCR) rate with nab-paclitaxel translated into a significantly improved invasive disease-free survival (iDFS) in patients with early BC as compared to that in sb-paclitaxel. It was also evaluated that peripheral sensory neuropathy (PSN) improved much faster under nab-paclitaxel 125 mg/m^2^ compared with nab-paclitaxel 148 mg/m^2^.

Schmid et al. [17] conducted a phase-III trial to study the effect of atezolizumab combined with nab-paclitaxel or placebo in combination with nab-paclitaxel on patients having untreated metastatic triple-negative BC. The two primary endpoints were progression-free survival (PFS) and overall survival, and the patients continued the administration until the occurrence of disease progression or an unacceptable level of toxic effects. They concluded that atezolizumab combined with nab-paclitaxel had shown continued PFS among patients having metastatic triple-negative BC in both the cases: intention-to-treat population and the programmed death ligand-1 (PD-L1)-positive subgroup.

Glassman et al. [21] examined and reported the post-approval safety and effectiveness of nanoliposomal irinotecan with fluorouracil/leucovorin (nal-IRI + 5-FU/LV) in advanced pancreatic cancer patients. This was the progression of their previous study where a phase-III randomized trial was conducted to demonstrate the efficacy of nal-IRI + 5-FU/LV for the treatment of advanced pancreatic cancer following progression on gemcitabine-based chemotherapy. This study demonstrated the safety and efficacy of nal-IRI + 5-FU/LV for the treatment of advanced pancreatic ductal adenocarcinoma (PDAC) following gemcitabine-based chemotherapy. Significant high overall survival (OS) was observed for nal-IRI + 5-FU/LV with an active combination of chemotherapy.

Current status of nanoparticles in lung cancer therapy

Despite numerous classes and types of anticancer drugs available or under study, the biggest challenging factor is to have such an anticancer agent that can specifically and exclusively target cancer cells only while reducing healthy tissue cytotoxicity. This has been the subject of evaluation in several clinical trials.

Spigel et al. [14] conducted a phase-III randomized clinical trial using nanoparticle albumin-bound paclitaxel in combination with carboplatin induction followed by nanoparticle albumin-bound paclitaxel for maintenance therapy in squamous non-small cell lung cancer (NSCLC) patients. Patients without disease progression after induction treatment were randomized to maintenance nanoparticle albumin-bound paclitaxel combined with best supportive care (BSC) or best supportive care alone. Though their study showed non-achievement of the primary endpoint, a trend in OS probability was observed, and maintenance therapy with nab-paclitaxel appears to be viable.

Shi et al. [15] conducted an open-label, randomized, multicenter, phase-III clinical trial study to compare the efficacy and safety between polymeric micellar paclitaxel (pm-Pac) plus cisplatin and solvent-based paclitaxel plus cisplatin as first-line treatment of advanced NSCLC. This study showed that the patients in the experimental arm had better objective response rates and PFS irrespective of the histological types. No significant difference was observed in the median OS and serious adverse events.

Weiss et al. [16] conducted a phase-II study of nab-paclitaxel for stage IV NSCLC patients who had been previously treated with a platinum doublet regimen and could have also received a PD-1 inhibitor. The study showed that nab-paclitaxel is a promising agent after disease progression with doublet chemotherapy in the geriatric population.

Socinski et al. [18] conducted a phase-III trial where untreated patients with stage IIIB to IV NSCLC were randomly assigned to receive nab-paclitaxel and carboplatin. The study showed that the administration of nab-PC as first-line therapy in patients with advanced NSCLC was efficacious and resulted in a significantly improved overall response rate (ORR) when compared to that of solvent-based paclitaxel (sb-paclitaxel).

In a study by Paz-Ares et al. [19], it was shown that the efficacy of nanoparticle-based paclitaxel is also retained in combination with pembrolizumab.

Yoneshima et al. [20] conducted an open-label, randomized, non-inferiority phase-III trial, comparing docetaxel or nab-paclitaxel. They concluded that the non-inferiority of nab-paclitaxel over docetaxel was confirmed concerning OS, PFS, and ORR. It was also evaluated that for patients with advanced NSCLC who have been previously treated with cytotoxic chemotherapy, nab-paclitaxel provides a clinically significant advantage in terms of effectiveness and tolerability.

In and Nieva [76] discussed the efficacy and toxicity of various nanoparticle chemotherapy agents. According to their review report, nanoparticle-based targeted chemotherapy has improved the treatment of NSCLC many folds when compared to conventional chemotherapy, but they still have certain limitations and need further advancements.

Table 4 represents a brief summary of recent clinical trial studies of nanomedicines for lung cancer.

**Table 4:** Summary of recent clinical trial studies of nanomedicines for lung cancer therapeutics NSCLC: Non-small cell lung cancer; DHA: Docosahexaenoic acid.

Active Drug	Nanomaterial Types	Types of Lung Cancer	Phase	Reference
Paclitaxel	Nanoparticle albumin-based (Nab)	Non-squamous NSCLC	III	[10,11]
		NSCLC	III	
Docetaxel (DTX)	Polymeric	Advanced or metastatic cancer, including lung cancer	I	[5,11]
		NSCLC	II	
Camptothecin	Polymeric	NSCLC	II	[11]
		Lung neoplasms, small cell lung cancer	I/II	[3]
	Cyclodextrin-based polymer	NSCLC	II	[5]
Hafnium oxide-containing nanoparticles		NSCLC	I	[11]
		Recurrent NSCLC	I	
Irinotecan	Polymeric	NSCLC	II	[11]
		Lung and breast cancer	II	
		Recurrent small cell lung carcinoma	II	
	Liposome	Small cell lung cancer	I/II/III	[3]
Paclitaxel	Polymeric micelle	NSCLC	II	[11]
		NSCLC	III	[3]
Cisplatin	Micellar	Solid tumors	I/II	[11]
SN-38	Polymeric micelle	Small cell lung cancer	II	[11]
Topotecan	Liposomal	Small cell lung cancer	II/III	[11]
DHA-bonded paclitaxel		NSCLC	III	[11]
Gold	Silica-Gold	Lung cancer	I	[3]
Docetaxel	Prostate-specific membrane antigen (PSMA) – targeting polymer	NSCLC	II	[5]

Many other randomized clinical trial studies involving the utilization of nanoparticles or nanomaterials in conjunction with FDA-approved anticancer medicines have been conducted and demonstrated in the past; however, as they were either pilot studies or under consideration studies, they are not mentioned or discussed in this article.

Limitations

Despite the promising preclinical results, the full translational potential of nanoparticles is yet to be achieved. This is primarily due to the challenges associated with reproducibility, scalability and potential toxicological and safety hazards, and specific targeting of the cancer cells [6]. Mitchell et al. [77] have emphasized the increased analysis of the interactions between nanoparticles of different morphologies and their interactions with various biological barriers within the human body. They have especially focused on the challenges associated with nanoparticle interactions inside the human body concerning the topo-morphology of nanoparticles. In lung cancer therapeutics, most of the nanoparticle-based drugs being developed are formulated for systemic intravenous or oral administration. This is associated with the problem of reduced bioavailability at the target site and toxicity due to the metabolism and excretion of nanomaterials in the mononuclear phagocytic system (MPS) and kidney. The side effects and metabolic profile of nanoparticles need further understanding, specifically the long-term physiological effects.

## Conclusions

Our narrative review shows that despite all of the advancements and development in the field of anticancer nanomedicines, the interaction of nanoparticles and the microenvironment in cancerous tissues requires further studies. This will not only help provide momentum to the translational research from the bench to bedside but also add insight into the efficacy results of various clinical trials. Transport of nanomaterials to tumor cells, targetability and cell specificity, release and pharmacokinetics of active ingredients, and excretion require "smart" and efficient drug-delivery systems. "Smart nanoparticles" incorporating versatile therapeutic agents (chemotherapeutic agents, immunotherapeutic agents, and radionuclides) have been attributed as “Trojan horses” in cancer therapeutics. The inherent advantage of "smart nanoparticles" will be reduced therapeutic resistance and enhancement of therapeutic ratio. Nanoparticles are also very promising as theragnostic agents and invaluable for future research in personalized medicine.
